# Characteristics of SOX9-positive progenitor-like cells during cholestatic liver regeneration in biliary atresia

**DOI:** 10.1186/s13287-022-02795-2

**Published:** 2022-03-21

**Authors:** Yuting Lin, Fang Zhang, Ludi Zhang, Lian Chen, Shan Zheng

**Affiliations:** 1 Department of Pediatric Surgery, Children’s Hospital of Fudan University, National Children’s Medical Center, Shanghai Key Laboratory of Birth Defect, and Key Laboratory of Neonatal Disease, Ministry of Health, 399 Wan Yuan Road, Shanghai, 201102 China; 2 State Key Laboratory of Cell Biology, CAS Center for Excellence in Molecular Cell Science, Shanghai Institute of Biochemistry and Cell Biology, Chinese Academy of Sciences, University of Chinese Academy of Sciences, Shanghai, 200031 China; 3 Department of Pathology, Children’s Hospital of Fudan University, National Children’s Medical Center, 399 Wan Yuan Road, Shanghai, 201102 China

**Keywords:** Biliary atresia, Reactive ductular cells, Hepatic progenitor cells, Liver progenitor-like cells, SOX9

## Abstract

**Background:**

The progression of Biliary Atresia (BA) is associated with the number of reactive ductular cells (RDCs) whose heterogeneity in origin and evolution in humans remains unknown. SOX9-positive liver progenitor-like cells (LPLCs) have been shown to participate in RDCs and new hepatocyte formation during cholestatic liver regeneration in an animal model, which implies the possibility that hepatocyte-reprogrammed LPLCs could be a source of RDCs in BA. The present study aimed to elucidate the characteristics of SOX9-positive LPLCs in BA for exploring new possible therapeutic targets by manipulating the bi-differentiation process of LPLCs to prevent disease progression.

**Methods:**

Twenty-eight patients, including 24 patients with BA and 4 patients with Congenital Choledochal Cyst as the control group, were retrospectively recruited. Liver biopsy samples were classified histologically using a 4-point scale based on fibrosis severity. LPLCs were detected by SOX9 and HNF4A double positive staining. Single immunohistochemistry, double immunohistochemistry, and multiple immunofluorescence staining were used to determine the different cell types and characteristics of LPLCs.

**Results:**

The prognostic predictors of BA, namely total bile acid (TBA), RDCs, and fibrosis, were correlated to the emergence of LPLCs. SOX9 and HNF4A double-positive LPLCs co-stained rarely with relevant markers of portal hepatic progenitor cells (portal-HPCs), including CK19, CK7, EPCAM, PROM1 (CD133), TROP2, and AFP. Under cholestasis conditions, LPLCs acquired superior proliferation and anti-senescence ability among hepatocytes. Moreover, LPLCs arranged as a pseudo-rosette structure appeared from the periportal parenchyma to the portal region, which implied the differentiation from hepatocyte-reprogrammed LPLCs to RDCs with the progression of cholestasis.

**Conclusions:**

LPLCs are associated with disease progression and prognostic factors of BA. The bipotent characteristics of LPLCs are different from those of portal-HPCs. As cholestasis progresses, LPLCs appear to gain superior proliferation and anti-senescence ability and continually differentiate to RDCs.

**Supplementary Information:**

The online version contains supplementary material available at 10.1186/s13287-022-02795-2.

## Introduction

Biliary atresia (BA) is a typical obstructive chronic cholestatic liver disease that has been the most common indicator for pediatric liver transplantation for several decades [1–3]. Diverse reactive ductular cells (RDCs) accompanying the cholestatic progression of BA are associated with the development of liver fibrosis and decreased native liver survival [4–6]. Though RDCs have been considered to participate in liver regeneration in animals [7–12], the heterogeneity underlying either the origin or evolution of RDCs in humans remains unknown.

Because RDCs are morphologically diverse, histopathologists speculate that RDCs could originate from many cell sources under certain conditions of injury [13–16]. Typical RDCs that are confined to the hepatic portal region with an intact arrangement of the lumen show positive staining for cholangiocyte and hepatic progenitor cell markers such as cytokeratin-19 (CK19), cytokeratin-7 (CK7), epithelial cell adhesion molecule (EPCAM), and sex-determining region Y-box 9 (SOX9). In contrast, atypical RDCs present discrete distribution with poorly defined lumen in parenchyma and co-stain with the hepatocyte markers albumin (ALB) and hepatocyte nuclear factor 4 alpha (HNF4A). Additionally, flattened cylinders of atypical RDCs are quite similar to hepatic muralia under three-dimensional anatomy observation [17]. These hepatobiliary bi-phenotypical atypical RDCs suggest the possibility of bipotent hepatic progenitor cells (HPCs) as their origin [18, 19]. Subsequent histological and animal lineage tracing studies indicated the bipotent status of the cells, which were the portal hepatic progenitor cells (portal-HPCs) derived from the Canal of Hering [19] and liver progenitor-like cells (LPLCs) derived from mature hepatocyte reprogramming [20, 21]. However, because the known HPC markers such as CK7, EPCAM, and SOX9 mostly overlap with markers of cholangiocytes, conventional single antibody staining using either of these antibodies cannot precisely differentiate these bipotent cells in human specimens [11, 22]. Thus, the relationship between portal-HPCs or LPLCs and patients’ prognosis predictors including atypical RDCs in BA needs further confirmation.

Oval cells as the most representative portal-HPCs that positively stain for the hepatoblast marker α-fetoprotein (AFP) were thought to differentiate into functional hepatocytes in hepatoxic rats [23, 24] and were found to be associated with the severity of some diseases in humans [25, 26]. Nonetheless, recent lineage tracing studies in mice have shown a limited contribution of oval cells to hepatocyte regeneration during the cholestatic condition [27–30]. On the other hand, SOX9-positive LPLCs subtly participated in new hepatocyte formation in chronic liver injury models [20, 21, 31]. LPLCs were found to be activated more frequently in the cholestatic injury model (DDC, BDL) than in the hepatocyte injury (CCl4, TAA) or hepatectomy model [21, 32–34]. During chronic cholestatic liver injury, hepatocytes resisted the environment by reprogramming to LPLC status within the periportal region and continued their differentiation into RDCs. As the injury subsided, LPLCs differentiated back to compensate for new hepatocytes. Moreover, those RDCs derived from LPLCs were proved to be functional and sustained after the cholestatic injury was reversed in a cholestatic disease Alagille syndrome mimic mice model [35], which is in accordance with trans-differentiation. Because lineage tracing techniques cannot be performed in humans, these cholestasis resistance properties of LPLCs and their contribution to liver regeneration in human cholestatic liver disease remain unknown.

In the present study, we determined that LPLCs were associated with disease progression and prognostic factors of BA. By evaluating the bipotent identity of LPLCs, our study elucidated the cholestasis resistance characteristics of LPLCs in BA, which could be the mechanism for the emergence of atypical RDCs. In future, interrupting hepatocyte reprogramming to LPLCs or the differentiation of LPLCs to RDCs may be a novel therapeutic approach to prevent disease progression in BA. Our study lays the foundation for this theory, which sheds new light on the treatment of BA.

## Materials and methods

### Human specimen collection

A total of 28 patients from Children’s Hospital of Fudan University, National Children’s Medical Center, Shanghai, China, were retrospectively analyzed. Eighteen liver biopsy samples were obtained from patients with extrahepatic BA during Kasai portoenterostomy (median age 1.9 ± 0.7 months, range 0–4 months). Another six liver biopsy samples were collected from patients with BA at the time of liver transplantation without previous Kasai portoenterostomy (median age 5.8 ± 1.1 months, range 4–7 months). For the control group, four liver biopsy samples obtained during the radical surgery of Congenital Choledochal Cyst in children aged 1 to 8 years were studied.

### Histopathological analysis

Liver fibrosis was assessed based on the modified Ishak staging system [36], which classifies liver fibrosis on a four-point scale with stage 4 being defined as cirrhosis. Histopathological slides were examined in a blinded and nonsequential manner and were re-evaluated by the same pathologist in this study.

### Immunohistochemistry and immunofluorescence analysis

The specimens were fixed with 4% paraformaldehyde (PFA) and embedded in paraffin after tissue processing. Standard hematoxylin and eosin staining was performed. The following primary antibodies were used in immunohistochemical staining: anti-CK19 (1:100; Gene Tech, Chengdu, China), anti-SOX9 (1:1000; Millipore), anti-HNF4A (1:400; Santa Cruz), anti-HepPar1 (1:10; Santa Cruz), anti-PKCζ (1:100; Santa Cruz), and anti-MRP2 (1:200; Gene Tech).

Single immunohistochemical staining was performed using the Elite ABC kit (Vector Laboratories). After deparaffinization, rehydration, and antigen retrieval, 2-µm-thick sections were blocked with 10% normal donkey serum for 1 h at room temperature and stained with primary antibodies. Biotinylated anti-rabbit (Vector Laboratories) and anti-mouse (Vector Laboratories) antibodies were used as secondary antibodies according to the manufacturer’s protocol. Biphenyl-3,3′,4,4′-tetrayltetraammonium tetrachloride (Dako) was used as a chromogen according to the manufacturer’s protocol. Double immunohistochemical staining was performed using the ImmPRESS Duet Double Staining Polymer Kit (HRP Anti-Rabbit IgG/AP Anti-Mouse IgG) (Vector Laboratories). Sections were pretreated in the same manner as that for single immunohistochemical staining as described above. After blocking, the sections were incubated with primary antibodies overnight, and the process was followed according to the manufacturer’s instructions.

For immunofluorescence analysis, the Opal™ 4-Color Manual IHC Kit (PerkinElmer) was used according to the manufacturer’s protocol. The following additional primary antibodies were used: anti-Ki67 (1:1000; Leica Biosystems), anti-CK7 (1:200; Abcam), anti-AFP (1:300; Dako), anti-CD133 (1:700; Cell Signaling), anti-p21 (1:1000; Abcam), and anti-PanCK (1:100; Abcam). All slides were incubated with primary antibody overnight, which was diluted with 10% normal donkey serum after 1-h serum blocking. After DAPI (4′,6-diamidino-2-phenylindole) staining, images were acquired using an Olympus BX51 microscope.

### Statistical analysis

Statistical analysis was performed using SPSS (Statistical Package for Social Sciences) software version 26.0. For the quantification of sections, four or more random periportal fields of each liver sample, unless otherwise specified, were imaged and then quantified using ImageJ software. Independent Kruskal–Wallis test was used to determine significant differences between the groups. Spearman’s correlation coefficient was used for the analysis of correlation. All data are presented as mean ± SEM. Differences were considered to be significant at *P* < 0.05. All experiments were performed with at least two technical replicates and repeated at least three times.

## Results

### LPLCs correlate with cholestatic progression in BA

**Fig. 1:**
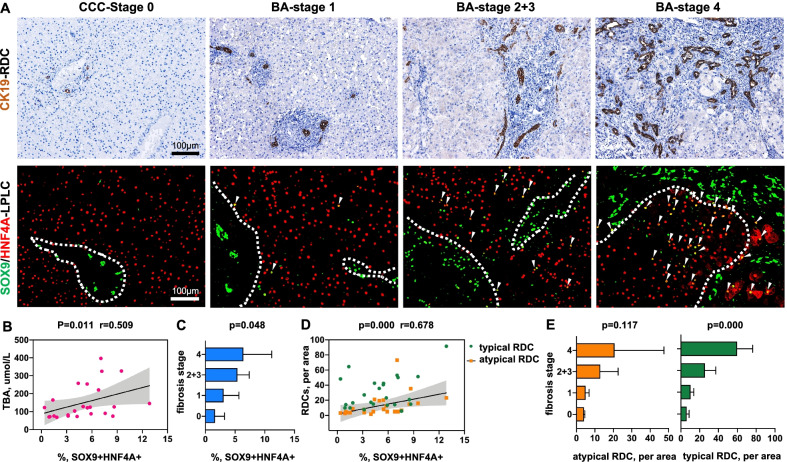
Correlation of LPLCs with cholestatic progression in BA. **A** Histological staining of LPLCs and RDCs for the different fibrosis progression groups. **B–D** Correlation analyses showing a positive relationship between LPLCs and cholestatic-related factors for BA: **B**, TBA; **C**, fibrosis; and **D**, RDCs. **E** Correlation analyses showing a correlation between the fibrosis stage and different types of RDCs. Atypical RDCs were inconsistently observed in the cirrhosis stage

In our study, hepatocytes, cholangiocytes, and LPLCs were marked with HNF4A + SOX9−, SOX9 + HNF4A−, and SOX9 + HNF4A+, respectively, following multiple immunofluorescence staining, and the cell count was estimated. RDCs were determined by immunohistochemical staining for CK19 in which typical and atypical RDCs were counted separately. Liver functions assessed by direct total bilirubin (DBIL), alkaline phosphatase (ALP), gamma-glutamyl transferase (GGT), aspartate aminotransferase (AST), alanine aminotransferase (ALT), and total bile acid (TBA) indices were significantly different between fibrosis severity (*P* < 0.05) (Table 1). The count of LPLCs and RDCs gradually increased as the disease progressed (Fig. 1A).

**Table 1:** Comparison of patient characteristics between the fibrosis groups

Variables	CCC Fibrosis stage 0 *n* = 4	BA Fibrosis stage 1 *n* = 6	BA Fibrosis stage 2 + 3 *n* = 12	BA Fibrosis stage 4 *n* = 6	*P* value
*Demographic data*					
Age, months	55 ± 28	1.4 ± 0.4	2.2 ± 0.7	5.8 ± 1.1	
Male gender, %	0 (0)	3 (50)	3 (50)	2 (33.3)	
*Laboratory data*					
DBIL, µmol/L	3.2 ± 0.8	127.5 ± 37.3	128.8 ± 15.5	173.5 ± 66.6	0.005*
ALP, IU/L	228.5 ± 67.8	591.5 ± 189.6	721.6 ± 180.3	431.5 ± 124.9	0.02*
GGT, IU/L	98.2 ± 91.9	191.4 ± 105.4	440.0 ± 319.3	501.7 ± 471.3	0.033*
ALT, IU/L	42.6 ± 32.2	144.7 ± 97.7	155.1 ± 51.4	304.7 ± 137.2	0.003*
AST, IU/L	35.85 ± 15.3	190.5 ± 85.1	242.5 ± 82.5	502.7 ± 229.8	0.001*
TBA, µmol/L	4.5 ± 5.5	77.7 ± 6.9	137.1 ± 65.6	246.8 ± 106.4	0.000*

For the BA sample, 18 specimens were obtained from the Kasai procedure, and 6 specimens were obtained from liver transplantation without Kasai surgery. Four Congenital Choledochal Cyst specimens were collected as controls

*P < 0.05

To elucidate the relationship between LPLCs and cholestatic progression in BA, we compared the number of LPLCs with factors associated with cholestasis, including liver function, fibrosis stage, and emergence of RDCs. The serum level of TBA in patients with BA significantly correlated with LPLC formation (*P* < 0.05) (Fig. 1B), but not with other liver function indices. The progression of fibrosis also showed a positive correlation with LPLCs (*P* < 0.05) (Fig. 1C). Regarding the emergence of RDCs, atypical RDC significantly correlated with the number of LPLCs (*P* < 0.001), while no relationship was observed between LPLCs and typical RDC (Fig. 1D). However, atypical RDC showed inconsistent emergence in the cirrhosis stage, while typical RDC showed a significant increase (Fig. 1E). Taken together, these results suggest an association between LPLCs and cholestatic progression in the liver with BA.

### Bipotent characteristics of LPLCs differ from those of portal-HPCs

As previous studies have elucidated the bipotent traits of LPLCs from HPCs, we questioned whether these bipotent cells belonged to the same cell clusters. To determine the identity of LPLCs, we performed multiple immunofluorescence staining of LPLCs with portal-HPC-related markers used in previous human and rodent studies.

**Fig. 2:**
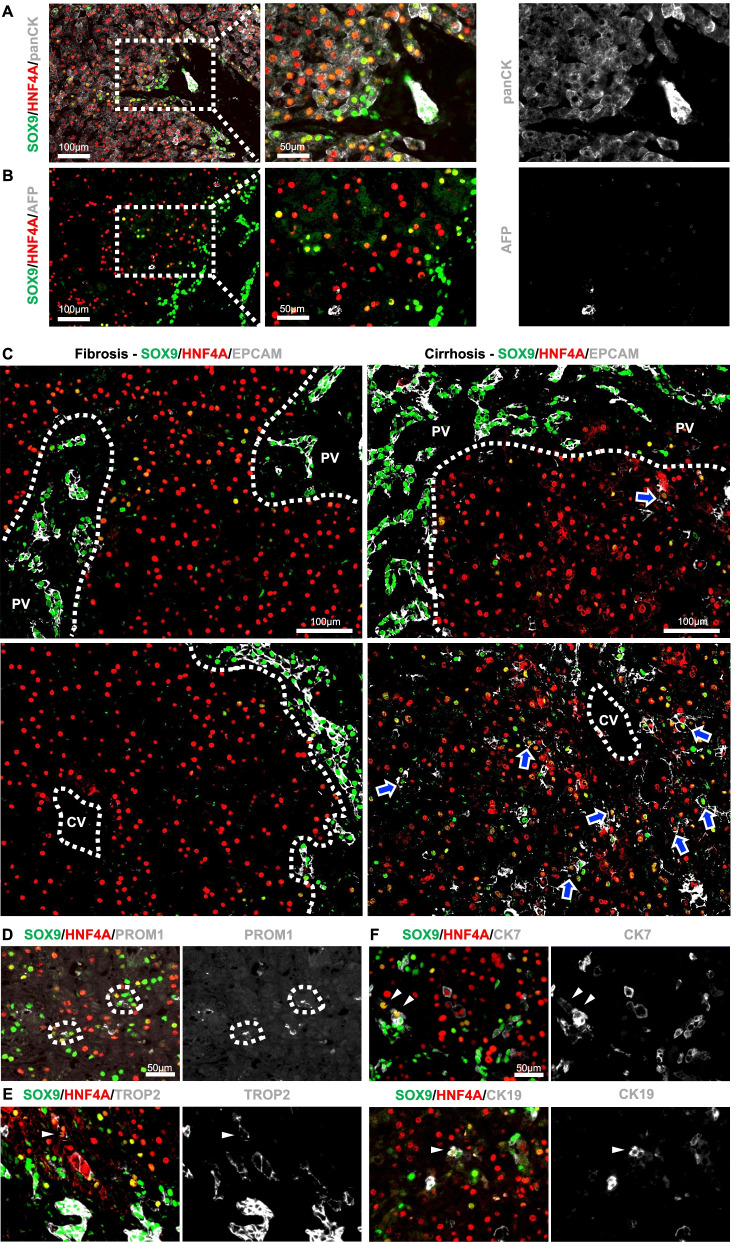
Bipotent characteristics of LPLCs differ from those of portal-HPCs. LPLCs co-stained with the relevant markers of portal-HPCs, including (**A**) epithelial cell marker (panCK), (**B**) hepatoblast marker (AFP), (**C–E**) hepatic progenitor markers (EPCAM, PROM1, and TROP2), and (**F**) RDCs markers (CK7 and CK19)

We first used an epithelial cell marker panCK to determine the epithelial features of LPLCs. As expected, panCK was expressed in hepatocytes, LPLCs, and cholangiocytes, but a stronger expression was observed in SOX9-positive cells (Fig. 2A). AFP as a hepatoblast marker is expressed during liver bud development, which normally shows a high level in a newborn’s liver and gradually decreases after 3–6 months of age. In our cohort, the age of all BA patients was under 6 months because of the difficulty in collecting specimens from older children. Therefore, the expression of physiological AFP might present overlapping with some hepatocytes in our patients. Indeed, our data showed that AFP co-stains with some hepatocytes alone in children within 6 months of age. However, AFP did not co-stain with LPLCs (Fig. 2B). The hepatic progenitor markers EPCAM seems to increasingly overlap with LPLCs as cholestasis progresses, especially in the adjacent central vein (Fig. 2C). Another hepatic progenitor markers PROM1 (CD133) showed low co-staining with LPLCs in our cohort (< 1%) (Fig. 2D). TROP2, a recently reported liver progenitor marker found in the scRNA-seq of the normal human liver [37, 38], also rarely overlapped with LPLCs (< 1%) (Fig. 2E). The most common markers that indicate RDCs, namely CK7 and CK19, rarely co-stained with LPLC (< 1%) (Fig. 2F). Interestingly, although the above-mentioned HPC and RDC markers were mostly expressed in the portal and periportal regions, we found that these markers can also be expressed in the adjacent central vein in two of our BA cases. In these cases, CK7 and CK19 almost completely overlapped with TROP2 in the portal region and the adjacent central vein (Additional file 1: Figure S1A-B). However, SOX9 is widely expressed beyond the portal region, which resulted in only partial overlap with TROP2 in the adjacent central vein but almost complete overlap with TROP2 in the portal region similar to CK7 and CK19 (Additional file 1: Figure S1C). In addition, co-staining of LPLC with portal-HPC and RDC markers was found mostly in these two cases. These zonation differences in the expression of HPC and RDC markers might further imply the heterogeneity of bipotent cells during cholestatic injury. Taken together, these observations indicate that the bipotent characteristics between LPLCs and portal-HPCs might be different.

### LPLCs exhibit superior proliferation and anti-senescence abilities as compared to SOX9-negative hepatocytes as cholestasis progressed

**Fig. 3:**
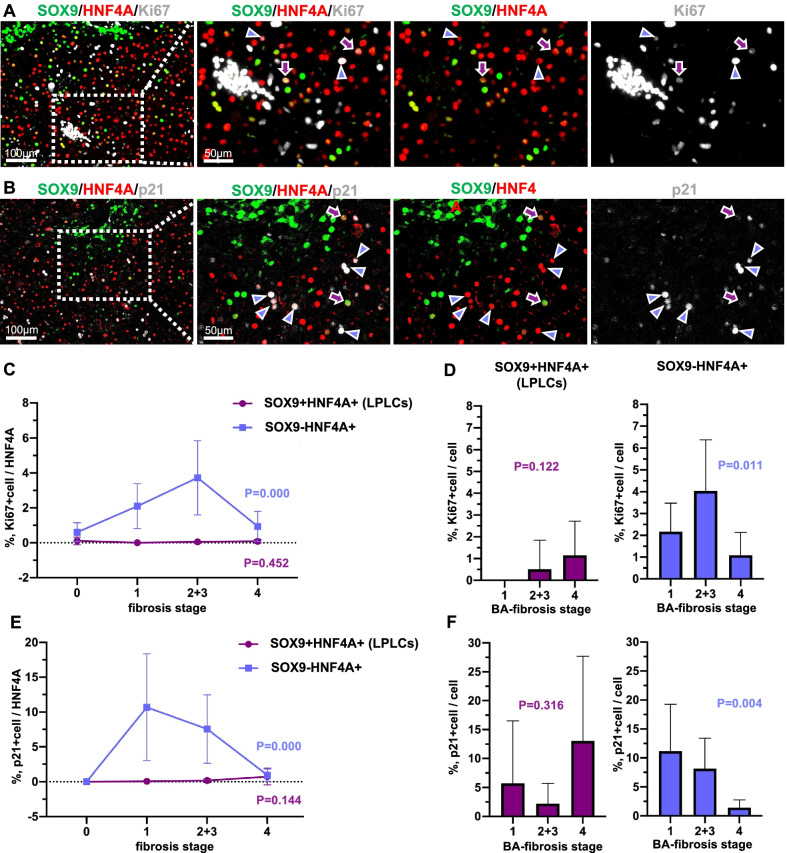
LPLCs exhibit superior proliferation and anti-senescence abilities as compared to SOX9-negative hepatocytes with the progression of cholestasis. Multiple immunofluorescence staining of LPLCs with (**A**) proliferation marker (Ki67) and (**B**) anti-senescence marker (p21). Overall percentage (**C**) and cell cluster percentage (**D**) of Ki67+ LPLCs and Ki67+ SOX9-negative hepatocytes in different fibrosis stages. Overall percentage (**E**) and cell cluster percentage (**F**) of p21+ LPLCs and p21+ SOX9-negative hepatocytes in different fibrosis stages

For better understanding of the unique characteristics of LPLCs during cholestatic injury, the proliferation (Ki67) (Fig. 3A) and anti-senescence (p21) (Fig. 3B) abilities of LPLCs and hepatocytes were evaluated.

During fibrosis stages 0–3, SOX9-negative hepatocytes showed increasing Ki67 positivity from 0.61 to 3.73%, while their Ki67 positive staining decreased to 0.94% in the cirrhosis stage. In contrast, the Ki67 positivity of LPLCs showed less fluctuation with the progression in cholestasis and was less than 0.2% in all fibrosis stages (Fig. 3C). However, the Ki67 positivity of the LPLC cluster gradually increased and reached 1.15% in the cirrhosis stage, which was higher than that of the SOX9-negative hepatocyte cluster (1.09%) (Fig. 3D).

The anti-senescence ability of SOX9-negative hepatocytes, as determined by p21 staining, increased to 10.68% in the initial stage of cholestasis (fibrosis stage1) and then gradually decreased as cholestasis progressed. In the cirrhosis stage (fibrosis stage 4), the p21 positivity of SOX9-negative hepatocytes was almost at the same level as that of LPLCs, i.e., 0.94% and 0.74%, respectively (Fig. 3E). Although the p21 positivity of LPLCs in each fibrosis stage was less than 0.2%, a prominent increase in the p21 staining of the LPLC cluster in fibrosis stage 4 might indicate that LPLCs undergo some changes in the cholestatic cirrhosis stage (Fig. 3F).

These data preliminarily show the superior proliferation and anti-senescence characteristics of LPLCs during cholestasis, although the proportion of LPLCs among overall hepatocytes was much less, resulting in no significant differences between the different fibrosis groups.

### LPLCs transform into RDCs within the periportal region during cholestasis progression

To investigate the cell plasticity of LPLCs during cholestasis progression, double immunohistochemical staining was performed for better morphological observation in our study. As both HNF4A and SOX9 are expressed in the nucleus, other markers that are specifically detected in the cytoplasm and apical membrane of hepatocytes (HepPar1 and MRP2) and the apical side of bile ducts (PKCζ) [32] were used to co-stain with SOX9.

**Fig. 4:**
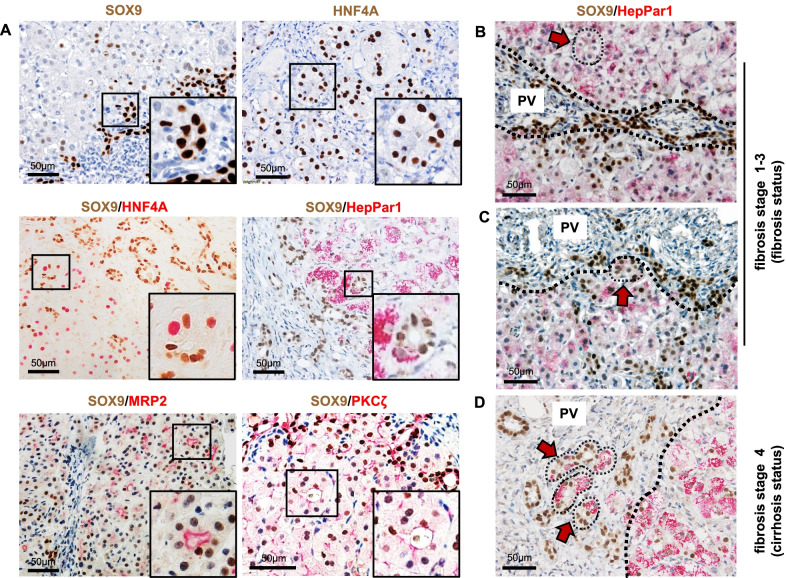
LPLCs differentiated into RDCs in the periportal region with the progression of cholestasis. **A** Pseudo-rosette formation in the periportal parenchyma appears to have bipotent characteristics during cholestatic liver damage, which co-stains with the LPLC markers SOX9 and HNF4A and with other hepatocyte and cholangiocyte markers, namely HepPar1, MRP2, and PKCζ. Pseudo-rosette hepatocytes (HepPar1-positive) appeared (**B**) in the periportal parenchyma with weak SOX9 staining and (**C**) in the adjacent portal region with increasing SOX9 staining and ductular-like structure in the fibrosis stage and (**D**) occurred inside the portal region with SOX9-positive ductular structure in the cirrhosis stage

In our study, LPLCs stained by SOX9 and HNF4A double-positive staining histologically appeared as a pseudo-rosette formation in periportal parenchyma. A pseudo-rosette formation is observed in dilated canaliculi surrounded with hepatocytes, and it is considered to be beneficial for draining excessive bile by forming new bile ductules adjacent in periportal parenchyma during cholestatic injury [39]. To further investigate LPLCs with a pseudo-rosette formation, we co-stained SOX9 with other hepatocyte markers, including HepPar1snd MRP2, as well as the bile duct marker PKCζ. The results showed SOX9 co-staining phenotype in the pseudo-rosette formation (Fig. 4A). Thus, we found that the bipotent phenotype of LPLCs partially overlapped with its pseudo-rosette formation characteristic.

Through histological observation from the fibrosis to cirrhosis stage, we also found that bi-phenotypical SOX9 co-staining HepPar1 cells emerged from the parenchyma to the portal region. During fibrosis stages 1–3 (fibrosis stage), the pseudo-rosette formation in the parenchyma showed positive HepPar1 staining but had weak SOX9 staining (Fig. 4B), while the formation showed higher SOX9 staining within the ductular-like structure when it appeared toward the boundary between the parenchyma and the portal region (Fig. 4C). In stage 4 fibrosis (cirrhosis stage), this SOX9 + HepPar1 + pseudo-rosette formation occurred inside the portal region and was lined with the ductular structure surrounded by collagen deposition (Fig. 4D). The change in the location of the SOX9-positive bi-phenotypical pseudo-rosette formation might suggest the possible trans-differentiation from hepatocytes to RDCs through the bi-phenotypic LPLC status.

## Discussion

In our present study, we clarified the correlation between LPLCs and disease progression of BA. Bipotent characteristics of LPLCs differ from portal-HPCs. Regarding cholestasis resistance characteristics, LPLCs present superior proliferation and anti-senescence ability and further differentiate into RDCs with the progression of cholestasis.

There have been sporadic reports that the transcription factor SOX9 is associated with the prognostic factors of BA [40, 41]; however, the identity of these SOX9-positive cells was unclear. As we have mentioned earlier, SOX9 as one of the HPC markers has a certain degree of overlap with cholangiocyte markers. To better elucidate SOX9-positive LPLCs, multiple immunofluorescence staining was used to determine SOX9 and HNF4A double-positive LPLCs in our study. We previously indicated that BA-related prognosis predictors such as TBA, RDCs, and fibrosis [5, 6, 42] are associated with the emergence of LPLCs. LPLCs correlated with atypical RDC, which agrees with previous literature that LPLCs could be a possible source for atypical RDC [43–45]. The progression of fibrosis seems to correlate with the emergence of LPLCs, while the number of active LPLCs was quite different in the six cirrhotic samples. We speculate that hepatocytes might undergo some irreversible damage in the cirrhosis stage, resulting in the deactivation of LPLCs.

LPLCs defined as Sox9 + Hnf4a + cells were first proposed as EpCAM- cells to highlight their hepatocyte lineage in mice [46]. Likewise, we first determined SOX9 and HNF4A double-positive cells and then co-stained them with other portal-HPC-associated markers. We found that LPLCs rarely show co-staining with the classic cholangiocyte and hepatic stem/progenitor markers CK19, CK7, TROP2, EpCAM, PROM1 (CD133), and AFP, even in cases of prominent emergence of LPLCs. TROP2-positive progenitor cells were selected from the EpCAM + population, which predicted its cholangiocyte lineage in human liver single-cell analysis [37, 38]. Among TROP2^low/–^, TROP2^int^, and TROP2^high^ compartments, the TROP2^low/–^ cluster upregulated the hepatocyte markers [38]. Based on our data, TROP2 rarely co-stained with LPLCs but showed a similar emerging trajectory to the ductular markers CK7 and CK19, which is in accordance with the above scRNA-seq finding. The heterogeneity of bipotent characteristics implies that LPLCs differ from portal-HPCs.

To elucidate the possible resistance characteristics of LPLCs during cholestatic injury, we assumed that SOX9-negative hepatocytes acquire superior proliferation and anti-senescence ability as they transform into SOX9-positive LPLCs. As expected, the Ki67 positivity of LPLCs increased gradually from the fibrosis stage to the cirrhosis stage, while the Ki67 co-staining was decreased in SOX9-negative hepatocytes in the cirrhosis stage. This finding suggests that SOX9-negative hepatocytes might not sustain its proliferation ability in a cirrhosis environment. p21 as a universal cell cycle inhibitor appeared to be more striking in our studies. p21 uniquely promotes G1 cell cycle arrest when the p53/p21 G1 checkpoint is triggered [47]. Thus, p21 is required for cellular response to stresses such as DNA damage and nutrient deprivation, which trigger the checkpoint and cause growth arrest. In our present study, p21 seemed to be activated in both SOX9-negative and SOX9-positive hepatocytes at the initial cholestatic progression stage in order to respond to cholestasis stress. As cholestasis became chronic, p21 co-staining hepatocytes gradually decreased. Hence, we presumed that the cell cycle might reactivate during the ongoing cholestatic injury; this assumption requires further studies for confirmation. Nevertheless, p21 co-staining SOX9-positive LPLCs emerged unexpectedly in the cirrhosis stage in their own cell cluster. The ability of p21 to promote the emergence of RDCs and the development of cirrhosis has been indicated previously [48]. Numerous studies have also shown that p21 is associated with tumorigenesis and metastasis of various cancers, including brain, lung, and colon cancer [49]. However, it remains to be further elucidated whether LPLCs are associated with hepatic tumorigenesis in liver cirrhosis.

During cholestatic injury, hepatocytes acquire SOX9 positivity and undergo ductal metaplasia into LPLCs [20, 32], which express mRNAs similar to those found in cholangiocytes rather than in hepatocytes [20, 50]. Subsequent lineage tracing studies revealed the bi-differentiation process of LPLCs during cholestatic injury [21, 31]. The animal experiments established the scientific theory that RDCs originate from hepatocyte-reprogrammed LPLCs during the disease progression of BA. Thus, we hypothesized that LPLCs differentiate continually to RDCs in order to escape from cholestatic injury. By co-staining SOX9 with other hepatocyte and ductular apical side markers, we found that the bipotent characteristics of LPLCs are consistent with those in the pseudo-rosette formation. Although the reason for this architectural appearance in chronic bile retention is not well understood, the pseudo-rosette formation is thought to provide some protection from injury caused by abnormal bile constituents [39], which is similar to our assumption. By observing the static location changes in the SOX9-positive rosette formation from fibrosis to cirrhosis, we found that the SOX9-positive rosette structure showed a gradual increase in the SOX9 expression level and changed to ductular-like structure from the periportal parenchyma to the portal region, which might imply the possibility that LPLCs differentiate to RDCs during cholestasis progression.

Though the rapid aggravation of liver fibrosis in BA is a consequence of multiple effects, it has proven to be associated with the emergence of numerous RDCs [4–6]. In contrast, along with the formation of RDCs is restricted in Alagille syndrome, another cholestatic liver disease with dysplasia of bile ducts, the progression of liver fibrosis appears slowly [4]. These phenomena indicate that RDCs as an indicator of liver regeneration in diverse diseases should be a key intervention target for future liver fibrosis therapy in BA. By disrupting the differentiation process of LPLCs, it is possible that the formation of RDCs will be reduced and further restrain the development of liver fibrosis.

## Conclusion

LPLCs as a possible source of atypical RDCs are associated with disease progression and prognostic factors of BA. Regarding bipotent characteristics, LPLCs are different from portal-HPCs. In terms of resistance characteristics during cholestatic injury, LPLCs show superior proliferation and anti-senescence ability and continually differentiate to new RDCs. Our present study revealed those characteristics of SOX9-positive LPLCs during cholestatic liver regeneration, which could provide a new avenue to manipulate the bi-differentiation process of LPLCs to prevent the emergence of RDCs and moderate the prognosis of BA.

## Supplementary Information

**Additional file 1: Fig. S1.** SOX9 expressed different zonation patterns from those of TROP2, CK7, and CK19 in cases with HPC and RDC markers in the adjacent central vein. TROP2 co-stained with (**A**) CK7 and (**B**) CK19 showed almost complete overlapping in the portal region and the adjacent central vein. (**C**) SOX9 showed almost overlapping with TROP2 only in the portal region but partial overlapping with TROP2 in the adjacent central vein

## Data Availability

The datasets used and/or analyzed during the current study are available from the corresponding author on reasonable request.

## Abbreviations

BA: Biliary atresia
RDCs: Reactive ductular cells
HPCs: Hepatic progenitor cells
LPLCs: Liver progenitor-like cells
SPSS: Statistical package for social sciences
CK19: Cytokeratin-19
CK7: Cytokeratin-7
EpCAM: Epithelial cell adhesion molecule
SOX9: Sex-determining region Y-box 9
ALB: Albumin
HNF4A: Hepatocyte nuclear factor 4 alpha
AFP: α-Fetoprotein
panCK: Pan cytokeratin
HepPar1: Hepatocyte specific antigen antibody
MRP2: Multidrug resistance-associated protein 2
PKCζ: Protein kinase Cζ
DDC: 3,5-Diethoxycarbonyl-1,4-dihydrocollidine
BDL: Bile duct ligation
CCl4: Carbon tetrachloride
TAA: Thioacetamide
PFA: Paraformaldehyde
DAPI: 4′,6‐Diamidino‐2‐phenylindole
DBIL: Direct total bilirubin
ALP: Alkaline phosphatase
γGT: Gamma-glutamyl transferase
AST: Aspartate aminotransferase
ALT: Alanine aminotransferase
TBA: Total bile acid
