# Supplementary figures and images for: Characteristics of SOX9-positive progenitor-like cells during cholestatic liver regeneration in biliary atresia
Source: Stem Cell Res Ther. 2022 Mar 21;13:114. doi: 10.1186/s13287-022-02795-2 (PMC8935712; doi:10.1186/s13287-022-02795-2)

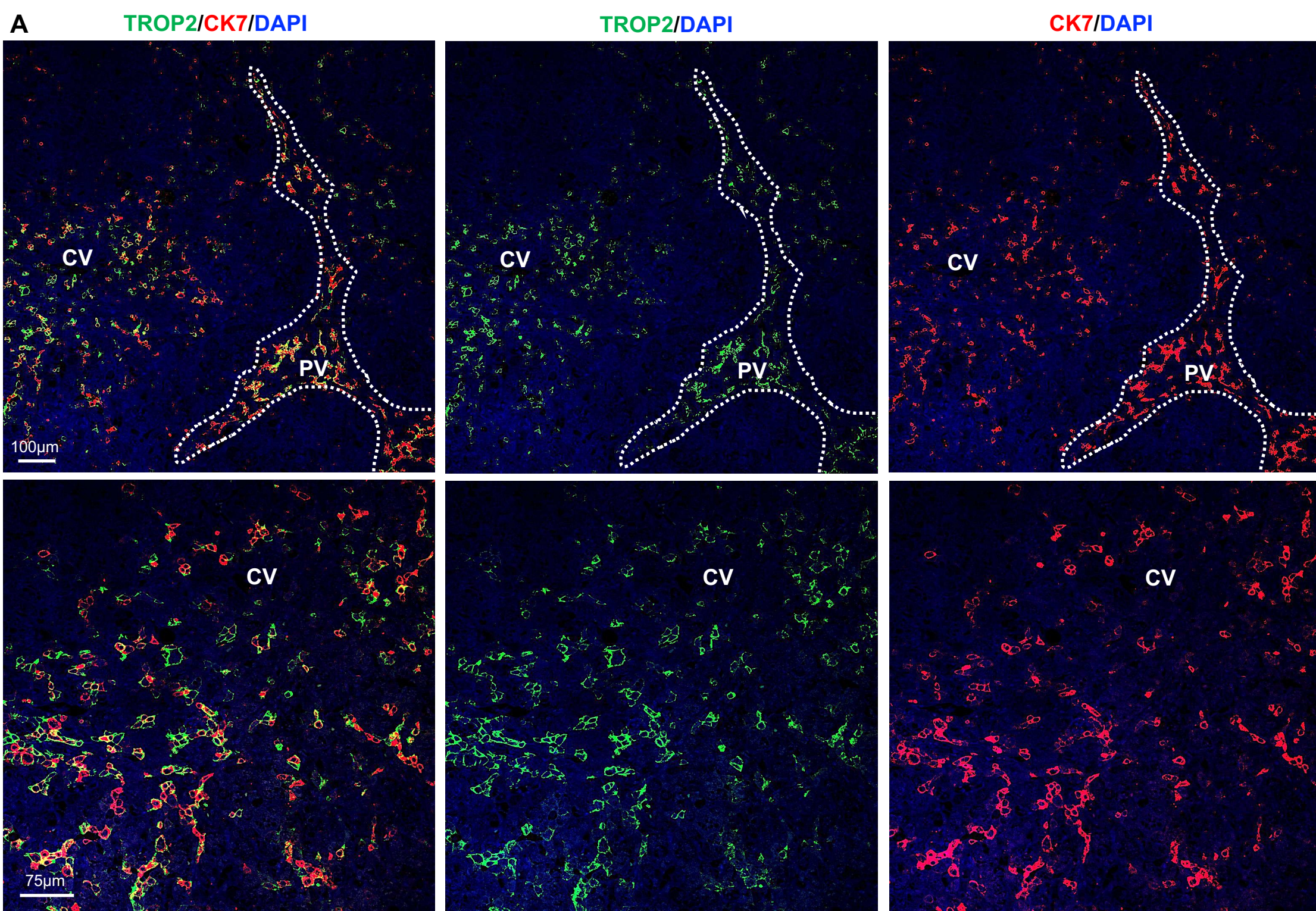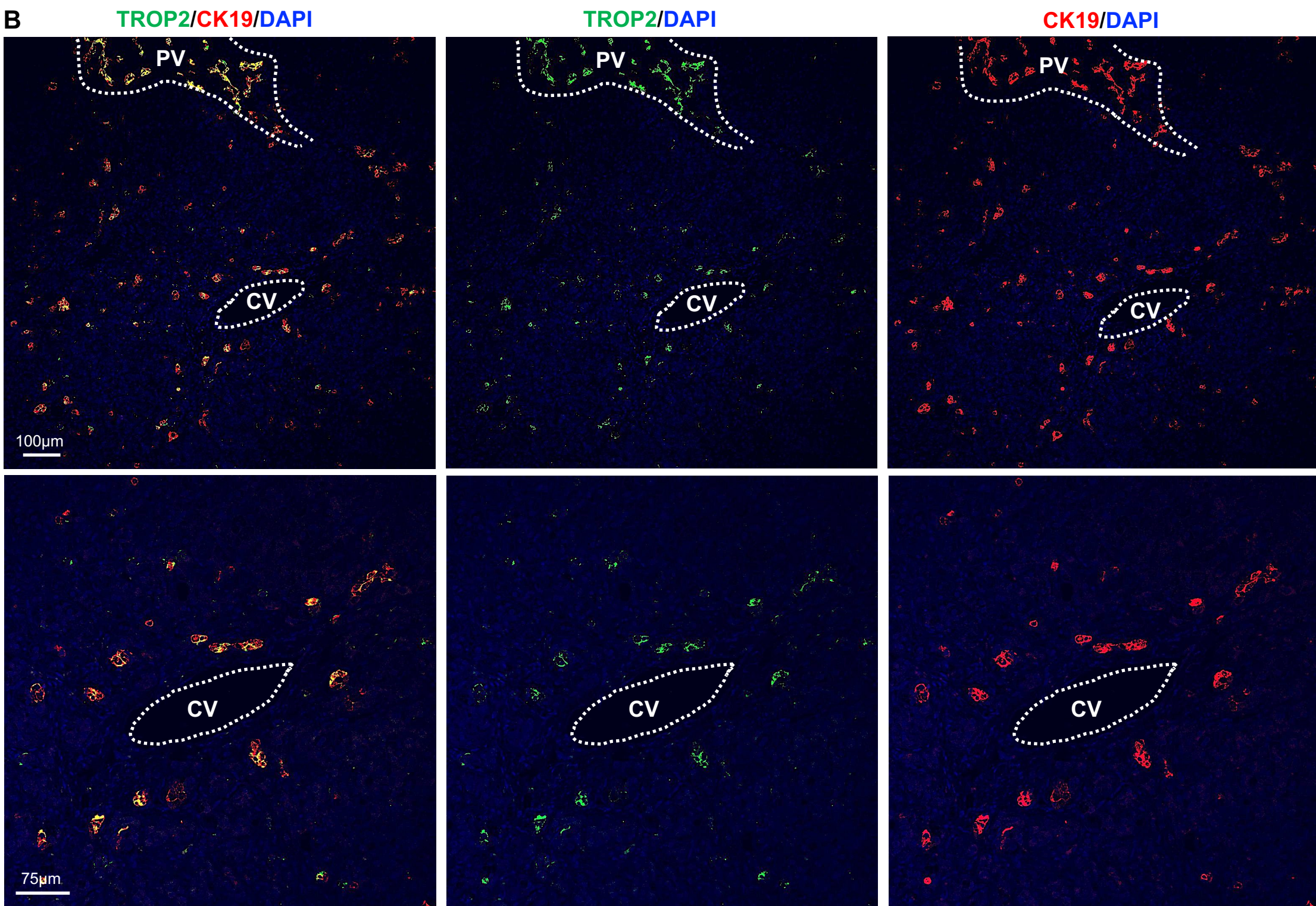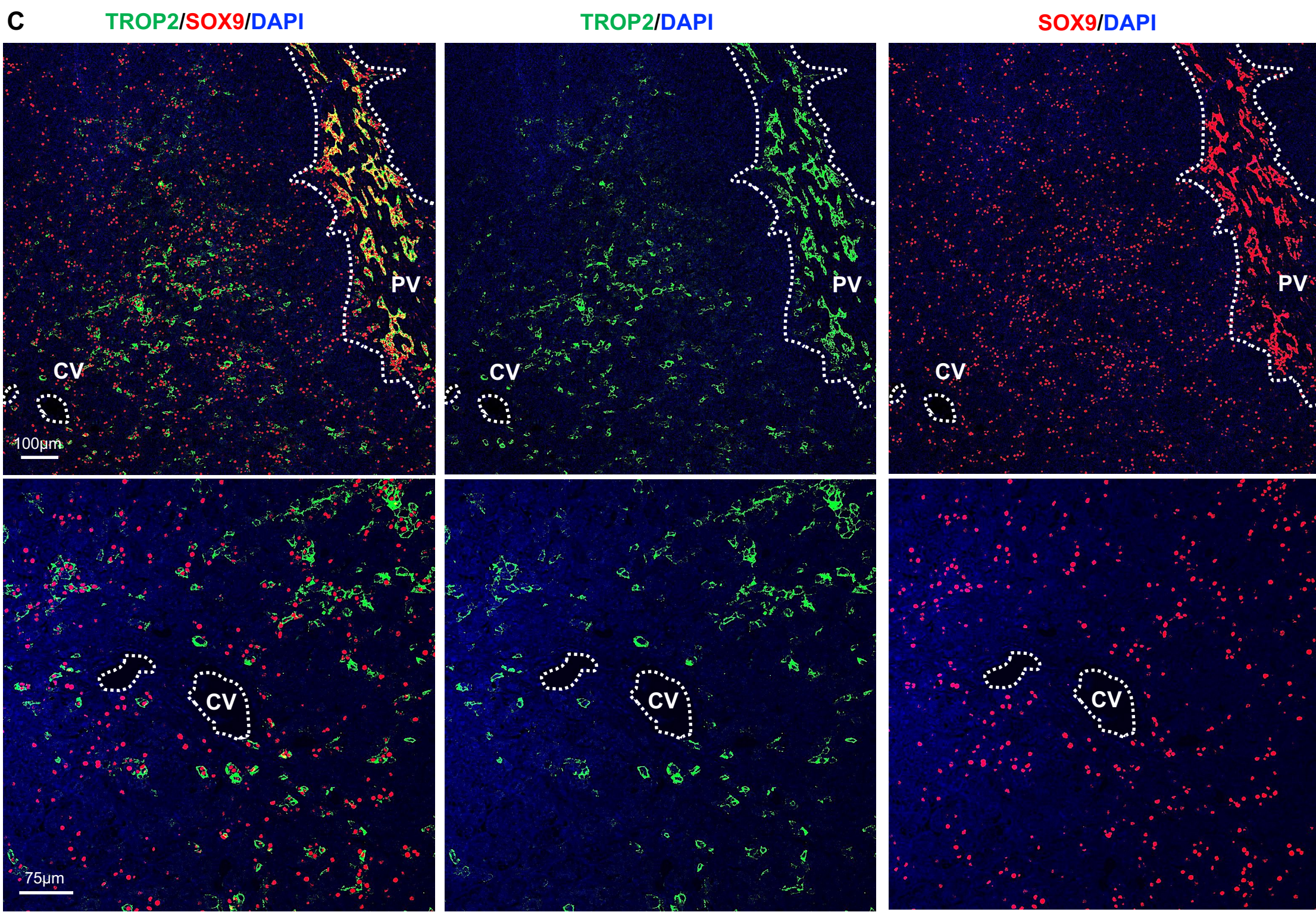

Supplement: Supplementary file 1 — Additional file 1: Fig. S1. SOX9 expressed different zonation patterns from those of TROP2, CK7, and CK19 in cases with HPC and RDC markers in the adjacent central vein. TROP2 co-stained with (A) CK7 and (B) CK19 showed almost complete overlapping in the portal region and the adjacent central vein. (C) SOX9 showed almost overlapping with TROP2 only in the portal region but partial overlapping with TROP2 in the adjacent central vein [file 13287_2022_2795_MOESM1_ESM.pdf]
